# Timing of definitive fracture fixation in patients with concomitant traumatic brain injuries – A systematic review of the literature by the IMPACT group

**DOI:** 10.1007/s00068-025-02981-w

**Published:** 2025-10-28

**Authors:** Felix Karl-Ludwig Klingebiel, Markus F. Oertel, Yannik Kalbas, Zsolt J. Balogh, Frank J. P. Beeres, Raul Coimbra, Christian Fang, Peter V. Giannoudis, Falco Hietbrink, Frank Hildebrand, Hayato Kurihara, Thomas Lustenberger, Ingo Marzi, Ruben Peralta, Shanmuganathan Rajasekaran, Emil H. Schemitsch, Heather A. Vallier, Boris A. Zelle, Hans-Christoph Pape, Roman Pfeifer

**Affiliations:** 1 Department of Trauma Surgery, University Hospital Zurich, University of Zurich, Zurich, Switzerland; 2 Harald-Tscherne Laboratory for Orthopaedic and Trauma Research, University Hospital Zurich, University of Zurich, Zurich, Switzerland; 3 Department of Neurosurgery, University Hospital Zurich, University of Zurich, Zurich, Switzerland; 4 Clinical Neuroscience Center, University Hospital Zurich, University of Zurich, Zurich, Switzerland; 5 Department of Traumatology, John Hunter Hospital and University of Newcastle, NSW Newcastle, Australia; 6 Department of Orthopaedic and Trauma Surgery, Lucerne Cantonal Hospital, Lucerne, Switzerland; 7 Riverside University Health System Medical Center and Loma Linda University School of Medicine, CA Loma Linda, USA; 8 Department of Orthopaedics and Traumatology, Li Ka Shing Faculty of Medicine, The University of Hong Kong, Hong Kong, Hong Kong; 9 Academic Department of Trauma and Orthopaedics, School of Medicine, University of Leeds, Leeds, UK; 10 NIHR Leeds Biomedical Research Centre, Chapel Allerton Hospital, Leeds, UK; 11 Department of Trauma Surgery, University Medical Centre Utrecht, Utrecht, The Netherlands; 12 Department of Orthopaedics, Trauma and Reconstructive Surgery, RWTH Aachen University Hospital, Aachen, Germany; 13 Emergency Surgery Unit, Fondazione IRCCS Ca’ Granda Ospedale Maggiore Policlinico, Milan, Italy; 14 Department of Trauma Surgery, Aarau Cantonal Hospital, Aarau, Switzerland; 15 Department of Trauma, Hand, and Reconstructive Surgery, University Hospital Frankfurt, Goethe University, Frankfurt/Main, Germany; 16 Department of Surgery, Trauma Surgery, Hamad Medical Corporation, Doha, Qatar; 17 Department of Surgery, Universidad Nacional Pedro Henriquez Urena, Santo Domingo, Dominican Republic; 18 Department of Orthopedics and Spine Surgery, Ganga Hospital, Coimbatore, India; 19 Department of Surgery, Division of Orthopaedic Surgery, University of Western Ontario, ON London, Canada; 20 Department of Orthopaedic Surgery, Cleveland Clinic Foundation, OH Cleveland, USA; 21 UT Health San Antonio, Department of Orthopaedics, TX San Antonio, USA

**Keywords:** Polytrauma, TBI, Timing of surgery

## Abstract

**Introduction:**

The timing of definitive fracture care in polytrauma patients remains a complex topic, especially in the presence of concomitant intracranial injuries, which often dictate surgical priorities. The International MultidisciPlinAry Consensus panel on polyTrauma (IMPACT) recently proposed recommendations on the timing of surgical interventions in polytrauma patients with competing priorities and identified some gaps in evidence. The aim of this study is to provide a systematic review of the scientific evidence on the timing of fracture fixation in patients with traumatic brain injuries (TBI).

**Material & methods:**

A systematic review on MEDLINE and EMBASE was performed, including original articles published between 2000 and 2024, comparing the outcomes of early (≤ 24 h) versus late (> 24 h) definitive fracture fixation in polytrauma patients with TBI. Demographic data, overall injury severity, and TBI severity of the respective cohorts were taken into consideration for qualitative analysis. Additionally, complication rates and outcomes were assessed.

**Results:**

A total of 9782 studies were identified. After applying the inclusion and exclusion criteria, 7 studies were finally included. Overall, significant heterogeneity was observed in the selection criteria, with some studies focusing on more severe and others on milder TBI, using different criteria. Overall, most studies provide evidence that early fracture fixation in patients with mild TBI might be beneficial for patients’ outcomes.

**Conclusions:**

Early definitive fracture fixation within 24 h should be attempted in polytrauma patients with concomitant mild TBI under specific conditions, which were previously defined by the IMPACT group. Furthermore, current evidence suggests that this approach is both safe and beneficial in a carefully selected patient population. In cases of moderate and severe TBI, decision-making is usually more difficult and complex, often requiring it to be individualized. However, we identified several gaps in the existing literature, particularly with regard to the heterogeneity of the available studies in terms of inclusion criteria, injury morphologies and investigated outcome parameters. Prospective or detailed registry studies are required to obtain further insight into this relevant topic.

**Supplementary Information:**

The online version contains supplementary material available at 10.1007/s00068-025-02981-w.

## Introduction

The timing of definitive fracture care in polytrauma patients remains a complex topic. Multiple factors must be considered, such as the patient’s physiological status and the overall injury pattern. Over the last several decades, clear physiologic stratification criteria for multiply injured patients into stable, borderline, unstable, and in extremis have been recommended to support surgical decision-making regarding the timing of surgical intervention [1].

Besides the patient’s physiology, the overall injury pattern plays a critical role in determining the timing of surgery and guiding surgical decision-making. Traumatic brain injury (TBI) is among the most common injuries in polytrauma patients [2]. The timing of definitive surgical fracture fixation in this patient cohort is under frequent scrutiny and is usually evaluated in close collaboration between neurosurgeons and orthopedic/trauma surgeons in clinical practice. Recent publications have demonstrated that damage control orthopedics (DCO) is frequently employed to reduce secondary injury to the brain in patients with TBI and an Abbreviated Injury Scale (AIS) of > 2 [3].

In 2024, the IMPACT group proposed 20 recommendations regarding the timing of definitive surgical fracture fixation with respect to concomitant injuries [4]. Overall, there seems to be evidence in the existing literature that patients generally benefit from definitive fixation within 24 h if the posttraumatic clinical condition allows it. The IMPACT group also identified certain conditions, including TBI, which could be considered as an indication for delayed definitive fracture care.

Although this review includes studies covering the full spectrum of TBI severity in polytrauma patients, the quality and consistency of the available evidence vary significantly across different severity groups. As a result, the discussion places greater emphasis on mild TBI. However, as far as possible, the recommendations encompass all severity levels, including the rationale for delayed surgery in patients with more severe TBI and/or specific intracranial pathologies.

The aim of this systematic review is to identify gaps in the available literature that need to be filled in order to improve our clinical decision-making and the timing of definitive fracture care in patients with TBI.

## Materials and methods

### Systematic review

The reporting of the systematic review adheres to the Preferred Reporting Items for Systematic Reviews and Meta-Analyses (PRISMA) guidelines (http://www.prisma-statement.org/). We performed a systematic review to identify all relevant publications on the timing of fracture fixation in trauma patients with concomitant TBI.

## Search strategy

The final systematic literature search was performed on August 18, 2024, using the Medline and EMBASE databases. The search covered studies published between January 1, 2000, and July 31, 2024. We used a combination of controlled vocabulary (MESH/Emtree-Terms) and regular search terms connected by Boolean operators. Truncation was used to capture plural forms and alternate spellings. Great care was taken to consider all relevant synonyms. Filters were applied to exclude inappropriate article types. The complete list of search terms is provided in the supplementary Material. In addition, we screened the reference lists of selected studies and related reviews for further eligible publications (referred to as “additional sources”).

## Extraction, screening and retrieval

Search results were extracted and organized in EndNote™ version 20 (Clarivate™, Philadelphia, USA). Articles were de-duplicated and then screened (title and abstract) independently by FKLK and YK. RP and MFO performed a cross-check of the extracted data. Any disagreement was resolved by a senior expert. The remaining articles were retrieved from the respective publishers through access to the university’s central library.

## Inclusion and exclusion criteria

Original articles and review papers written in English were assessed for inclusion. Articles were included if they reported on the timing of fracture care in multiply injured adult patients, focusing on the outcomes of patients with concomitant TBI.

Guidelines, commentaries, conference abstracts, correspondences, expert opinions, editorials, letters, book chapters, and experimental studies (in vitro or in vivo) were excluded. Further exclusion criteria included studies involving pediatric populations and combat-related trauma, as well as those with insufficient characterization of injury patterns or injured body regions, and those that did not specifically address the timing of fracture care.

## Qualitative assessment

From the included publications, patient cohort-specific information was extracted, including sample size, timing cut-offs, overall injury severity (ISS), and TBI-specific injury severity (AIS), as well as the respective treated fracture location. Furthermore, complication and outcome rates, including mortality, acute respiratory distress syndrome (ARDS), pneumonia, hospital length of stay (HLOS), and neurological outcomes, were assessed. The collated data were then tabulated and analyzed qualitatively.

In a previous manuscript, our group discussed and evaluated the definitions of early” and “late” surgical care based on an extensive number of publications [5]. A cut-off was established at 24 hours and was therefore also be used in this article.

If the timing cut-offs were established not only at 24 h in the included publications, but also at earlier or later points, these were grouped into the ≤ 24 h or > 24 h category, respectively, and the statistics were recalculated based on the available data. Patients receiving non-operative treatments were excluded from the study.

As the cohort characteristics (i.e., TBI-specific inclusion criteria, timing cut-offs) and the assessed outcome parameters exhibited variation across the studies, and there was a lack of uniformity in the reporting of statistical results (i.e., usage of mean/median, SD/IQR), a quantitative comparison such as a meta-analysis could not be performed.

The level of evidence of included publications was rated using the GRADE [6].

(Grading of Recommendations, Assessment, Development, and Evaluation) approach by two authors independently. Any discrepancies were resolved by a third senior author.

## Results

### Characteristics

**Fig. 1:**
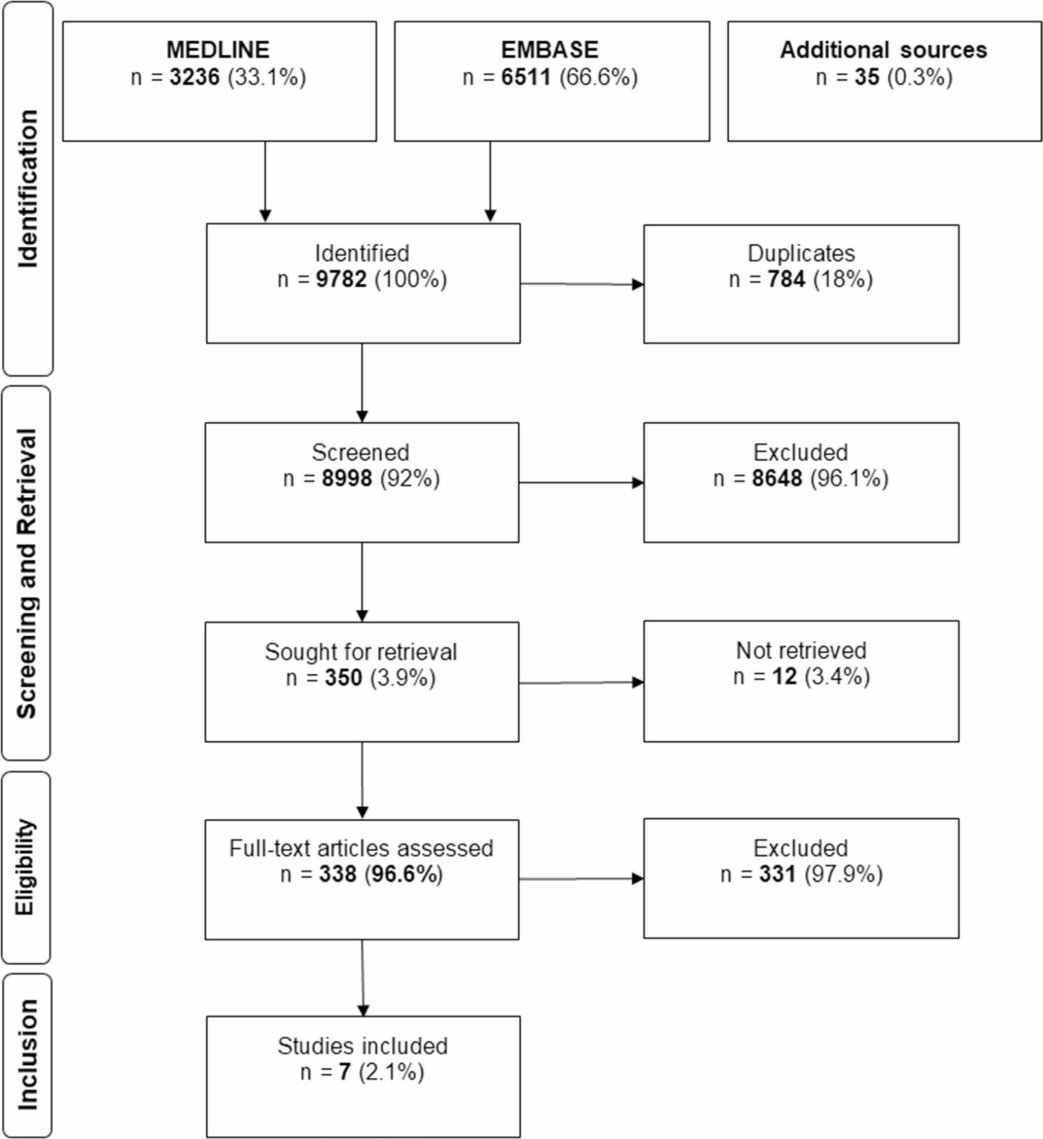
Flowchart of article inclusion

A total of 9782 publications were identified in the literature search of Medline and EMBASE. After de-duplication and application of inclusion and exclusion criteria during the initial screening process, 338 articles were included in the full text assessment phase. Of these, only seven studies focused on the patient’s outcome regarding the timing of definitive fracture care and were included in the study (Fig. 1).

Figure 1: Flowchart of article inclusion.

Six of the included papers had a retrospective design [7–12], while one was prospective [13]. The cohort size in the publications ranged from 96 to 3069 patients. Three publications directly compared outcomes of definitive fracture fixation ≤ 24 h vs. >24 h [9, 11, 12]. Two studies used multiple time cut-offs, ranging from 12 to 24 h up to 120 h [7, 10]. Two other studies compared definitive vs. temporary surgical fixation, yet al.so with a cut-off at 24 h [8, 13]. The studies applied different inclusion criteria to their cohorts regarding TBI severity, ranging from only mild TBI with intracranial trauma sequelae, as defined by the AIS scale, to TBI of all severities. Only three studies reported the specific type of intracranial bleeding and injury morphology [9, 12, 13]. Four studies focused exclusively on femoral shaft fractures [7, 8, 10, 11], two on long bone fractures in the lower extremities [12, 13], and one on all surgeries except neurosurgical procedures [9]. Three studies concluded that early surgery was beneficial [7, 11, 13], two were neutral with a tendency to favor late surgery [9, 12], and two supported late surgical stabilization [8, 10] (Table 1).

**Table 1:** Overview of included studies; fracture fixation timing groups were grouped into ≤ 24 h vs. >24 h

Authors	Year	Analysis	*N*	Groups	LoE	Timing cutoff	Type of TBI evaluated (Stage/classification)	Report bleeding	Fracture care	Favors
Brundage et al. [7]	2002	Retrospective	512	< 24 h vs. 24–48 h vs. 48–120 h vs. >120 h vs. nonoperative	2 A	Respective groups	Only AIS Head ≥ 2	No	Femoral shaft fractures	Early
Scalea et al. [8]	2004	Retrospective	324	EF vs. IMN	2B	24 h	All	No	Femoral shaft fractures	Late
Wang et al. [9]	2007	Retrospective	96	Early vs. Late	2B	24 h	Patients with craniotomy excluded	Yes	All except neurosurgical	None
Morshed et al. [10]	2009	Retrospective	3069	< 12 h vs. 12–24 h vs.24–48 h vs. 48–120 h vs. >120 h	2 A	Respective groups	All	No	Femoral shaft fractures	Late
Nahm et al. [11]	2011	Retrospective	248	Early vs. Late	2B	24 h	All	No	Femoral shaft fractures	Early
Ghneim et al. [13]	2023	Prospective	358	EF vs. IMN vs. ORIF	2 C	24 h	Mild-severe TBI + AIS Head > 2	Yes	Lower extremity long bone fractures	Early
Yu et al. [12]	2023	Retrospective	181	Early vs. Late	2 A	24 h	Only mild TBI + AIS Head ≥ 2	Yes	Lower extremity long bone fractures	None

*ORIF* Open Reduction and Internal Fixation, *IMN* Intra-Medullary Nailing, *EF* Early fixation, External fixation = Delayed fixation, *AIS* Abbreviated Injury Score, *LoE* Level of Evidence (according to GRADE), *N* Number of patients included, *TBI* Traumatic brain injury

## Outcomes

Most studies (*n* = 6) report the ISS/New Injury Severity Score (NISS) and AIS for the head, with significant differences between the study cohorts. The ISS ranged from 16.8 (median) to 39.85 (mean) between the cohorts, and the AIS was reported to be between 1.72 ± 1.66 and 5 (median). The absence of uniformity in the reporting process is a cause for concern. A qualitative visual inspection reveals a significant discrepancy in the severity of TBI-specific injuries reported by the studies. Two studies reported the Glasgow Coma Scale (GCS) [14] at discharge [7, 12], one study reported the revised Rancho Los Amigos Scale (RLAS-R) [13], and another study investigated neurophysiological and functional scores in their patient cohort [9]. Three studies did not report any functional neurological outcome parameters [8, 10, 11].

Mortality was the most frequently reported outcome (*n* = 6), followed by HLOS (*n* = 5), pneumonia (*n* = 4), and ARDS (*n* = 3) (Table 2). Overall, complication rates tended to be lower in the early intervention groups, which also exhibited a lower severity of cerebral injury, compared to the late intervention groups.

**Table 2:** Overview of injury severity and outcome parameters of the included studies; calculations were adjusted to the new pooled groups, early vs. late

Authors	*N* (E vs. L)	ISS (mean ± SD)	Severity TBI (AIS)	Mortality (*n*/%)	ARDS (*n*/%)	Pneumonia (*n*/%)	HLOS (days) (*n*/%)	Reported neurological outcome
Brundage et al. [7]	283 vs. 95	N/A	E: 3.8 vs. L: 3.05 (mean)	E: 11 (3.9%) vs. L: 95 (4.2%)	E : 21 (7.4%) vs. L : 14 (14.7%)	E: 39 (14%) vs. L: 21 (22.1%)	E: 15.5 vs. L: 20.47 (mean)	GCS
Scalea et al. [8]	281 vs. 43	E:16.8 vs. L = 26.8 (median)	AIS ≥ 3 = E: 41 (15%) vs. L: 24 (56%)	E: 1 (< 1%) vs. L: 4(9%)	N/A	N/A	E: 5.7 (3.0–10.1.0.1) vs. L: 17.5 (8.8–26.5) (median/IQR)	N/A
Wang et al. [9]	43 vs. 53	E: 29 vs. L:29 (median)	E: 5 vs. L:5 (median)	E: 2 (4.7%) vs. L: 2 (3.8%)	E: 5% vs. L: 2%	E: 23% vs. L: 42%	E: 13 vs. L: 17 (median)	Multiple neuropsychological and functional scores
Morshed et al. [10]	2299 vs. 770	E: 27.32 ± 8.82 vs. L: 31.29 ± 11.36 (NISS)	E: 1.72 ± 1.66 vs. L: 2.21 ± 1.80 (mean ± SD)	E: 75 (3.26%) vs. L: 33 (4.29%)	N/A	N/A	N/A	N/A
Nahm et al. [11]	199 vs. 49	E: 30.68 vs. L: 39.85	N/A	E: 3 (1.5%) vs. L: 3 (6.12%)	E: 6 (3.02%) vs. L: 3 (6.12%)	E: 39 (13.78%) vs. L: 11 (22.45%)	N/A	N/A
Ghneim et al. [13]	313 vs. 45	E: ISS < 16: 6(4%), 16–25: 50(29%), > 25: 115 (67%) vs. L: ISS < 16: 5(9%), 16–24: 7(16%), > 25%: 33(75%)	E: AIS 2–3 (n/%): 96(56%), 4–5: 77 (44%) vs. L: AIS (n/%): 2–3: 24(58%), 4–5: 18 (42%)	E: 23 (7.35%) vs. L: 8 (17.7%)	N/A	N/A	E: 17.55 (9.55–29.55) vs. L: 23 (14–38) (median/IQR)	RLAS-R Scale
Yu et al. [12]	78 vs. 103	E: 21.9 vs. L: 22.2 (mean)	E = 2.5 (± 0.6) vs. L = 2.7 (± 0.9) (mean ± SD)	N/A	N/A	E = 5 (6.4%) vs. L = 8 (7.8%) (="Pulmonary complications”)	E = 15.4 (± 10.0) vs. L = 16.9 (± 10.7)	GCS

*AIS* Abbreviated Injury Score, *ARDS* Acute Respiratory Distress Syndrome, *E* Early, *HLOS* Hospital Length Of Stay, *ISS* Injury Severity Score, *N/A* Not Available, *NISS* New Injury Severity Score, *TBI* Traumatic Brain Injury, *L* Late, *GCS* Glasgow Coma Scale, *RLAS-R* Rancho Los Amigos Cognitive Scale

## Discussion

The main challenge in the management of patients with concurrent TBI and fractures requiring definitive surgical fixation is to identify those who will benefit most from early fracture fixation, while avoiding harm to others. This remains extremely difficult to study based on the currently available literature. As highlighted in this review, four of the seven included studies excluded either severe or mild traumatic brain injuries, primarily based on a combination of GCS and head AIS.

The key finding that can be drawn from this literature review is as follows:

Most studies suggest that early definitive fracture fixation, usually within 24 h, may be attempted in polytrauma patients with concomitant mild TBI, provided that specific clinical conditions are met (Table 3).

**Table 3:** Recommendations on the timing of definitive fracture care by the IMPACT group published in “early major fracture care in polytrauma—priorities in the context of concomitant injuries: A Delphi consensus process and systematic review” [4]

No	Statement
	Traumatic Brain Injury
1	In patients with mild TBI (GCS ≥ 13) and initial head CT without evidence of acute intracranial trauma sequelae with adequate respiratory and hemodynamic parameters, early (< 24 h) definitive fracture fixation of isolated major fractures is recommended.
2	In patients with mild TBI (GCS ≥ 13) and initial head CT without evidence of acute intracranial trauma sequelae with adequate respiratory and hemodynamic parameters, early (< 24 h) definitive fracture fixation of multiple major fractures is permissible as long as the patient remains physiologically stable during serial reassessment. If the patient becomes unstable, consider DCO.
3	In mild TBI patients (GCS ≥ 13) with acute intracranial trauma sequelae findings on initial head CT, early (< 24 h) definitive fixation of major fractures is permissible after exclusion of significant intracranial pathologies and lack of further progression on follow-up head CT.
4	TBI patients should NOT undergo definitive fixation of major fractures in the presence of intracranial hypertension (ICP > 20 mmHg), deterioration of neurological status, progression of the initial trauma sequelae findings on head CT, hemodynamic and/or respiratory instability, and coagulopathy.
5	In patients with significant TBI, delayed definitive fracture fixation of major fractures is acceptable in patients with stable GCS, without deterioration of neurological status and findings in the follow-up head CT, or stable ICP (ICP ≤ 20 mmHg) and CPP (> 60–70 mmHg) in patients with invasive neuromonitoring.

There are challenges when using GCS in the polytrauma patient. Although widely adopted, the GCS is by far not an optimal scale as it may be altered for many reasons such as pre-existing neurological deficits, transient loss of consciousness, sedative medications, and drug or alcohol intoxication. Therefore, the score may not always be exclusively related to the extent of intracranial injuries, nor reflect the true extent of intracranial injury in all cases [15]. A recent study reported only a weak correlation between the GCS and the head AIS, highlighting the limitations of relying solely on the GCS for decision-making [16]. Additionally, the initial GCS scores in elderly patients may deteriorate over time after injury due to age-related brain atrophy, which creates increased space between the skull and brain parenchyma. This allows for the accumulation of larger volumes of extra-axial hemorrhage and brain swelling before significant neurologic symptoms or marked changes in the GCS scores become apparent [17]. Therefore, the timing of GCS deterioration can vary or be delayed, despite similar trauma severity and morphology. This may lead to an initial underestimation of the true extent of brain injury in some cases. Despite these limitations, the GCS remains widely used to classify mild, moderate, and severe TBI.

Similarly, stratification by the head AIS has multiple limitations. First, this parameter is calculated retrospectively and may not accurately reflect the acute situation in the trauma bay [18]. In addition, the interobserver reliability of this scoring system has been criticized in the past [19]. The term ‘mild TBI’ as defined in the AIS catalogue (2005) is paraphrased as ‘commotio cerebri’, and ranges from AIS 1 to AIS 2, with AIS 2 being the most common [20]. In cases of traumatic brain injury (TBI) accompanied by a brief loss of consciousness, the Abbreviated Injury Scale (AIS) assigns a rating of 2, equating its severity to that of subarachnoid or minor sub- or epidural bleeding. When the loss of consciousness lasts 60 min or more, the AIS is raised to 3, corresponding to a brain edema or an ischemic brain infarct. This should be considered at least a moderate TBI [21]. The ACRM definition of mild TBI, as endorsed by the WHO, uses a 30-minute loss of consciousness cut-off for the identification of this condition, contrasting with the 60-minute threshold applied by the AIS [22]. Nevertheless, there appears to be a consensus among experts that a prolonged loss of consciousness may be associated with increased concussion severity, although the precise cut-off point remains to be definitely established.

Only a few publications have investigated different neuropsychological or functional outcomes and their dependence on the timing of definitive fracture fixation. Although these are outcome measures of great interest, different assessment scales were used in some studies, while others did not report them at all. None of the included studies specifically reported on the extent of the initial blood loss and the results of diagnostic computed tomography (CT) scans, both of which are crucial factors in surgical decision-making. Therefore, the IMPACT group developed a set of recommendations for the management of fractures in patients with concomitant TBI.

The primary goal of managing patients with TBI is to prevent secondary brain injury. In addition to the primary insult itself, factors such as severe bleeding, hypoxia, hypotension, and coagulopathy increase the risk of secondary brain damage by contributing to cerebral ischemia or intracranial bleeding. Therefore, in patients with TBI, DCO is often used to minimize the risk of secondary brain injury. However, based on the results of the current literature review, there seems to be a benefit of early definitive fixation for a specific subgroup of TBI patients. Despite inconsistencies in the varying definitions and inclusion criteria regarding the TBI severity across the included articles, there is also evidence to suggest that patients with mild TBI may benefit from early fracture care. This is further supported by the results of a recent publication suggesting that early definitive fixation in stable TBI patients not undergoing neurosurgical intervention was not associated with increased mortality [23].

Therefore, in hemodynamically stable patients with mild TBI and without relevant intracranial trauma sequelae on neuroimaging or further deterioration, early fracture fixation should be performed. If the patient’s physiological condition worsens, a transition to DCO is recommended.

If there is evidence of acute intracranial trauma sequelae on neuroimaging in a patient with mild TBI, progression of the initial trauma sequelae needs to be ruled out before performing definitive fracture fixation. In cases with signs of intracranial hypertension, deterioration of the neurologic status, progression of initial trauma sequelae on follow-up imaging, or worsening of the patient’s physiological condition, early definitive fixation should be avoided. Delayed definitive fracture fixation can be performed in patients with relevant TBI if the GCS, neurologic status, and intracranial pressure remain stable, and no new pathological findings or progression of preexisting trauma sequelae are observed on follow-up neuroimaging. A relevant TBI underlies an individual evaluation of each patient, but most often includes moderate and severe TBI with relevant neurological impairment or findings in neuroimaging.

It is evident that the existing literature is subject to selection bias, as patients with more severe injuries are less likely to receive definitive fracture care, which is often delayed in accordance with the principle of damage control [24]. However, from an ethical perspective, conducting randomized controlled trials is not feasible due to the absence of clinical equipoise.

In patients with relevant intracranial trauma sequelae, initial definitive fracture care is of lower priority. First, neurosurgical interventions such as placement of invasive neuromonitoring devices, external ventricular drainage, or evacuation of intracranial hematomas may be required emergently [25]. Additionally, the patient’s hemodynamic status is of utmost importance, as intracranial pressure (ICP) and cerebral perfusion pressure (CPP) must be maintained within stable ranges and thus require close monitoring and management [26]. Since changes in the patient’s blood pressure might have a direct effect on the ICP and CPP, management of non-life-threatening injuries should be delayed due to the risk of associated blood loss. Most studies exploring the relationship between the hemodynamic status, neurological deterioration, potential blood loss, and the timing of fracture fixation included primarily patients with femur fractures. This may have occurred because internal fixation by means of intramedullary reaming is known to affect CPP due to a reduction in mean arterial pressure (MAP) [27]. Therefore, most temporary fracture fixation procedures follow the DCO principles [28]. A recent study investigated the ICP and CPP thresholds below which patients would qualify for safe definitive surgery (SDS) and their respective stratification into stable, borderline, unstable, and in extremis categories. An ICP < 15mmHg and CPP > 60-70mmHg in the absence of a midline shift on head CT would be indicative of a stable TBI patient [29]. In addition, resuscitation with a specific focus on adequate coagulation control is essential after trauma and during surgery to prevent further bleeding and its associated additional consequences [30].

As outlined by the IMPACT group, early definitive fracture care generally appears beneficial for polytrauma patients with concomitant TBI. This statement is confirmed by our systematic review, as early definitive fracture fixation seems to be beneficial for patients with mild TBI. The IMPACT group recommended considering the patient’s physiological condition, presence of additional injuries, neurological status deterioration, follow-up cranial neuroimaging results, and ICP and CPP monitoring values before deciding on definitive fracture fixation (Table 5). Definitive fracture fixation and reconstruction can be attempted if the patient’s physiology and clinical status have improved.

For surgical decision-making, the assessment of brain injury severity, specific brain injury patterns, and neurologic status, as well as the values of intracranial pressure monitoring, must be determined. Bleeding progression requires close observation, particularly in those undergoing general use of anticoagulation medications and in patients with trauma-induced coagulopathy. These factors must be considered when deciding the timing and type of fracture fixation.

### Limitations

The study is limited by the number and overall comparability of the included studies. Although injury severity was commonly reported using the ISS/NISS and AIS, most studies did not report the specific intracranial injury pattern (i.e., bleeding type) or progression on follow-up neuroimaging. Therefore, the conclusion should not be generalized to all patients with intracranial trauma. Rather, it suggests that a specific subgroup of patients—outlined in the IMPACT group recommendations—may benefit from early surgical intervention [4]. Due to the heterogeneity across the included studies, especially regarding TBI severity with varying definitions (AIS/GCS-based), findings and conclusions cannot be generalized to all patient cohorts. Only a minority of the publications report neurological outcomes, which – particularly in patients with mild TBI – surely would be a very relevant parameter. Conclusions were therefore drawn based on the available data and on parameters correlating with neurological outcomes such as HLOS. Overall, the evidence – especially regarding specific outcomes – remains limited. The association between early fracture fixation and reduced mortality may also be confounded by the initial surgical decision-making, as patients at risk are generally more likely to undergo delayed definitive fixation.

## Conclusion

Early definitive fracture fixation within 24 h should be attempted in polytrauma patients with concomitant mild TBI under specific conditions, which were previously defined by the IMPACT group. Furthermore, current evidence suggests that this approach is both safe and beneficial in a carefully selected patient population. In cases of moderate and severe TBI, decision-making is usually more difficult and complex, often requiring it to be individualized. However, we identified several gaps in the existing literature, particularly with regard to the heterogeneity of the available studies in terms of inclusion criteria, injury morphologies and investigated outcome parameters. Prospective or detailed registry studies are required to obtain further insight into this relevant topic.

## Supplementary Information

Below is the link to the electronic supplementary material.

Supplementary Material 1

## Data Availability

No datasets were generated or analysed during the current study.

## Abbreviations

AIS: Abbreviated Injury Scale
ACRM: American Congress of Rehabilitation Medicine
ARDS: Acute Respiratory Distress Syndrome
CI: Confidence Interval
CPP: Cerebral Perfusion Pressure
CT: Computed Tomography
DCO: Damage Control Orthopedics
GCS: Glasgow Coma Scale
GRADE: Grading of Recommendations Assessment, Development and Evaluation
HLOS: Hospital Length of Stay
ICP: Intracranial Pressure
IQR: Interquartile Range
ISS: Injury Severity Score
MAP: Mean Arterial Pressure
NISS: New Injury Severity Score
PRISMA: Preferred Reporting Items for Systematic Reviews and Meta-Analyses
RLAS-R: Rancho Los Amigos Scale - Revised
SD: Standard Deviation
SDS: Safe Definitive Surgery
TBI: Traumatic Brain Injuries
WHO: World Health Organization
