# Supplementary material for: Timing of definitive fracture fixation in patients with concomitant traumatic brain injuries – A systematic review of the literature by the IMPACT group
Source: Eur J Trauma Emerg Surg. 2025 Oct 28;51(1):308. doi: 10.1007/s00068-025-02981-w (PMC12568869; doi:10.1007/s00068-025-02981-w)
Supplement: Supplementary file 1 — Supplementary Material 1 [file 68_2025_2981_MOESM1_ESM.docx]

**Supplementary Information S1: Search strategy (performed 18.08.2024)**

**MEDLINE**

(("acute"[Title/Abstract] OR "definit*"[Title/Abstract] OR "delay*"[Title/Abstract] OR "early"[Title/Abstract] OR "emergen*"[Title/Abstract] OR "immediat*"[Title/Abstract] OR "late"[Title/Abstract] OR "manage*"[Title/Abstract] OR "primary"[Title/Abstract] OR "prior*"[Title/Abstract] OR "sequenc*"[Title/Abstract] OR "strateg*"[Title/Abstract] OR "timed"[Title/Abstract] OR "timing"[Title/Abstract] OR "urgent"[Title/Abstract]) AND (("Fracture Fixation"[MeSH Terms] OR ("closed red*"[Title/Abstract] OR "CRIF"[Title/Abstract] OR "fixat*"[Title/Abstract] OR "fract*"[Title/Abstract] OR "instrum*"[Title/Abstract] OR "nail*"[Title/Abstract] OR "open red*"[Title/Abstract] OR "ORIF"[Title/Abstract] OR "osteosyn*"[Title/Abstract] OR "plat*"[Title/Abstract] OR "screw*"[Title/Abstract] OR "stabil*"[Title/Abstract])) AND ("acetab*"[Title/Abstract] OR "ankl*"[Title/Abstract] OR "elbow"[Title/Abstract] OR "femor*"[Title/Abstract] OR "femur"[Title/Abstract] OR "hip"[Title/Abstract] OR "humer*"[Title/Abstract] OR "knee"[Title/Abstract] OR "lower extr*"[Title/Abstract] OR "pelvi*"[Title/Abstract] OR "radi*"[Title/Abstract] OR "shoulder"[Title/Abstract] OR "spin*"[Title/Abstract] OR "tibia*"[Title/Abstract] OR "upper extr*"[Title/Abstract] OR "wrist"[Title/Abstract])) AND ("Brain Injuries"[MeSH Terms] OR "subarachnoid*"[Title/Abstract] OR "subdural*"[Title/Abstract] OR "epidural*"[Title/Abstract] OR ((((("aneuris*"[Title/Abstract] OR "bleed*"[Title/Abstract] OR "contus*"[Title/Abstract] OR "compress*"[Title/Abstract]) AND "haemato*"[Title/Abstract]) OR "hemato*"[Title/Abstract] OR "injur*"[Title/Abstract] OR "traum*"[Title/Abstract]) AND "brain"[Title/Abstract]) OR "cereb*"[Title/Abstract] OR "crani*"[Title/Abstract] OR "head"[Title/Abstract] OR "intracr*"[Title/Abstract] OR "parench*"[Title/Abstract]))) AND (clinicalstudy[Filter] OR clinicaltrial[Filter] OR comparativestudy[Filter] OR controlledclinicaltrial[Filter] OR equivalencetrial[Filter] OR evaluationstudy[Filter] OR multicenterstudy[Filter] OR observationalstudy[Filter] OR randomizedcontrolledtrial[Filter] OR validationstudy[Filter])

Filter applied: 2000-2024

**EMBASE**

('acute':ab,ti OR 'definit*':ab,ti OR 'delay*':ab,ti OR 'early':ab,ti OR 'emergen*':ab,ti OR 'immediat*':ab,ti OR 'late':ab,ti OR 'manage*':ab,ti OR 'primary':ab,ti OR 'prior*':ab,ti OR 'sequenc*':ab,ti OR 'strateg*':ab,ti OR 'timed':ab,ti OR 'timing':ab,ti OR 'urgent':ab,ti)

**AND**

('fracture fixation'/exp/mj

**OR**

('closed reduc*':ab,ti OR 'crif':ab,ti OR 'fixat*':ab,ti OR 'fract*':ab,ti OR 'instrum*':ab,ti OR 'nail*':ab,ti OR 'open red*':ab,ti OR 'orif':ab,ti OR 'osteosyn*':ab,ti OR 'plat*':ab,ti OR 'screw*':ab,ti OR 'stabil*':ab,ti)

AND

('acetab*':ab,ti OR 'ankl*':ab,ti OR 'elbow':ab,ti OR 'femor*':ab,ti OR 'femur':ab,ti OR 'hip':ab,ti OR 'humer*':ab,ti OR 'knee':ab,ti OR 'lower extr*':ab,ti OR 'pelvi*':ab,ti OR 'radi*':ab,ti OR 'shoulder':ab,ti OR 'spin*':ab,ti OR 'tibia*':ab,ti OR 'upper extr*':ab,ti OR 'wrist':ab,ti))

**AND**

(('brain injury'/exp/mj OR 'subarachnoid*':ab,ti OR 'subdural*':ab,ti OR 'epidural*':ab,ti)

**OR**

(('aneuris*':ab,ti OR 'bleed*':ab,ti OR 'contus*':ab,ti OR 'compress*':ab,ti OR 'devasc*':ab,ti OR 'haemato*':ab,ti OR 'hemato*':ab,ti OR 'injur*':ab,ti OR 'traum*':ab,ti)

AND

('arter*':ab,ti OR 'brain':ab,ti OR 'cereb*':ab,ti OR 'crani*':ab,ti OR 'head':ab,ti OR 'intracr*':ab,ti OR 'kidney':ab,ti OR 'parench*':ab,ti OR 'vasc*':ab,ti)))

Filter:

[2000-2024]/py AND ('case control study'/de OR 'clinical article'/de OR 'clinical study'/de OR 'clinical trial'/de OR 'cohort analysis'/de OR 'comparative study'/de OR 'controlled clinical trial'/de OR 'controlled study'/de OR 'cross sectional study'/de OR 'longitudinal study'/de OR 'major clinical study'/de OR 'multicenter study'/de OR 'observational study'/de OR 'phase 1 clinical trial'/de OR 'prospective study'/de OR 'randomized controlled trial'/de OR 'retrospective study'/de) AND [article]/lim AND [english]/lim AND [abstracts]/lim AND [clinical study]/lim AND [embase]/lim
